# Comparison of phacotrabeculectomy versus phacocanaloplasty in the treatment of patients with concomitant cataract and glaucoma

**DOI:** 10.1186/1471-2415-13-1

**Published:** 2013-01-29

**Authors:** Juliane Matlach, Florentina Joyce Freiberg, Swetlana Leippi, Franz Grehn, Thomas Klink

**Affiliations:** 1 Department of Ophthalmology, University of Wuerzburg, Josef-Schneider-Str. 11, D-97080, Wuerzburg, Germany

**Keywords:** Non-penetrating glaucoma surgery, Phacotrabeculectomy, Phacocanaloplasty, Canaloplasty, Trabeculectomy

## Abstract

**Background:**

Cataract and glaucoma are both common comorbidities among older patients. Combining glaucoma surgery with minimal invasive phacoemulsification (phaco) is a considerable option to treat both conditions at the same time, although the combination with filtration surgery can produce a strong inflammatory response. Combined non-penetrating procedures like canaloplasty have shown to reduce intraocular pressure (IOP) comparable to trabeculectomy without the risk of serious bleb-related complications. The purpose of this retrospective study was to compare the outcomes of phacotrabeculectomy and phacocanaloplasty.

**Methods:**

Thirty-nine eyes with concomitant cataract and glaucoma who underwent phacotrabeculectomy (n = 20; 51.3%) or phacocanaloplasty (n = 19; 48.7%) were included into this trial on reduction of IOP, use of medication, success rate, incidence of complications and postsurgical interventions. Complete success was defined as IOP reduction by 30% or more and to 21 mmHg or less (definition 1a) or IOP to less than 18 mmHg (definition 2a) without glaucoma medication.

**Results:**

Over a 12-month follow-up, baseline IOP significantly decreased from 30.0 ± 5.3 mmHg with a mean of 2.5 ± 1.2 glaucoma medications to 11.7 ± 3.5 mmHg with a mean of 0.2 ± 0.4 medications in eyes with phacotrabeculectomy (P < .0001). Eyes with phacocanaloplasty had a preoperative IOP of 28.3 ± 4.1 mmHg and were on 2.8 ± 1.1 IOP-lowering drugs. At 12 months, IOP significantly decreased to 12.6 ± 2.1 mmHg and less glaucoma medications were necessary (mean 1.0 ± 1.5 topical medications; P < .05). 15 patients (78.9%) with phacotrabeculectomy and 9 patients (60.0%) in the phacocanaloplasty group showed complete success according to definition 1 and 2 after 1 year (P = .276). Postsurgical complications were seen in 7 patients (36.8%) of the phacocanaloplasty group which included intraoperative macroperforation of the trabeculo-Descemet membrane (5.3%), hyphema (21.1%) and bleb formation (10.5%). Although more complications were observed in the phacotrabeculectomy group, no statistically significant difference was found.

**Conclusions:**

Phacocanaloplasty offers a new alternative to phacotrabeculectomy for treatment of concomitant glaucoma and cataract, although phacotrabeculectomy yielded in better results in terms of IOP maintained without glaucoma medications.

## Background

There has been a widespread shift towards the performance of combined surgeries to treat both glaucoma and cataract in one surgical setting [1-3]. In fact, incisional glaucoma surgery in patients with medically uncontrolled glaucoma may induce an earlier cataract development [4,5] while performing the cataract surgery alone may not appropriately lower intraocular pressure (IOP) in glaucoma patients [6,7]. Simultaneous filtration surgery and clear corneal phacoemulsification in elderly patients with both cataract and glaucoma aims at reducing surgical trauma resulting from two separate surgeries [1]. Unfortunately, a combined procedure may induce an inflammatory response resulting in scarring of the filtering bleb with unsatisfactory IOP reduction. Since filtering glaucoma surgery requires a more intensive postoperative care than non-penetrating glaucoma methods [8-10] some patients may benefit from a less invasive glaucoma procedure. Several non-filtering alternatives such as deep sclerectomy [11,12] and viscocanalostomy [13,14] combined with cataract surgery have been proposed for the management of concomitant cataract and glaucoma. Although these methods may not achieve similar low intraocular pressures as trabeculectomy, their advantage could be the reduction of potential complications associated with filtering blebs.

Canaloplasty is a new non-penetrating glaucoma method using a 360° microcatheter dilation of Schlemm canal and a tension suture to restore the normal aqueous outflow pathway through Schlemm canal and the collector channels. It has a lower complication rate than combined procedures using a filtering bleb and provides a better IOP reduction when performed in combination with cataract surgery [15-17].

In this retrospective comparative study, the safety and efficacy profile of cataract surgery combined with trabeculectomy as compared to canaloplasty was investigated in adult patients with glaucoma and cataract. To date, a comparative analysis of phacotrabeculectomy and phacocanaloplasty has not yet been reported.

## Methods

### Patient selection

The study protocol was approved by the ethics committee of the University of Wuerzburg, Germany. Clinical records of consecutive patients who underwent phacocanaloplasty at the University Eye Hospital Wuerzburg, Germany between June 2008 and December 2010 were retrospectively analyzed. Data of consecutive patients who were treated with phacotrabeculectomy and mitomycin C (MMC) between May 2006 and December 2010 were reviewed over an extended period of time to have a comparable sample size. Phacotrabeculectomy or phacocanaloplasty was performed in patients with visually significant cataract and medically uncontrolled primary or secondary (pseudoexfoliative) open-angle glaucoma. Since cataract extraction will open up the angle, three patients with open-angle glaucoma but slightly narrow angles were also included. In these cases, no goniosynechiae were seen on gonioscopy prior to glaucoma surgery. Patients with juvenile, traumatic, neovascular or uveitic glaucoma were excluded from the study. A total of 24 phacocanaloplasty surgeries in 22 patients and 28 phacotrabeculectomy procedures in 28 patients were performed during the above mentioned time periods. Nineteen eyes (48.7%) of 19 patients undergoing phacocanaloplasty and 20 eyes (51.3%) of 20 patients undergoing phacotrabeculectomy were included according to the above mentioned inclusion and exclusion criteria. All phacocanaloplasty surgeries were performed by one single surgeon. Phacotrabeculectomy was performed by 3 experienced glaucoma surgeons of whom one also did all phacocanaloplasty surgeries.

### Evaluation of outcomes

General baseline information for each patient included age, gender, localisation of eye undergoing surgery, type of glaucoma, and ocular medication. Preoperatively, all patients received a standard ophthalmic examination including best corrected visual acuity (BCVA) measurement converted to the logarithm of the minimum angle of resolution (logMAR), IOP measurement using Goldmann applanation tonometry, angle grading by gonioscopy, slit lamp biomicroscopy of the anterior segment, and indirect ophthalmoscopy of the optic nerve head with documentation of size of optic disc, disc cupping, presence of an optic disc notch or splinter hemorrhage, and peripapillary atrophy.

### Surgical technique

#### Phacotrabeculectomy

A 6 o´clock corneal traction suture was placed and a fornix-based conjunctival flap was created. Different concentrations of MMC (0.2 to 0.5 mg/ml) were used according to the estimated individual risk for future bleb scarring. 4 sponge pieces (2 x 8 mm) soaked in MMC were placed under the conjunctival flap for 3 minutes followed by irrigation of the site with 30 ml balanced salt solution (BSS). MMC 0.2 mg/ml was used in the majority of patients (n = 14, 70.0%). In 6 eyes (30.0%) MMC 0.5 mg/ml was applied. Thereafter, a rectangular scleral flap measuring 4 × 3 mm was dissected. Before continuing filtration surgery, a standard phacoemulsification (phaco) technique with a temporal clear corneal incision and posterior chamber intraocular lens implantation was performed (two-site phacotrabeculectomy). Afterwards, a trabeculectomy of 0.8 × 2 mm was performed followed by an iridectomy. The scleral flap was secured with single sutures placed at both corners of the scleral flap and two additional sutures if needed. Finally, a 10.0 nylon running mattress suture was used to close the conjunctiva.

### Phacocanaloplasty

After conjunctival incision at the 12 o´clock position, a superficial parabolic scleral flap measuring 5 × 5 mm was dissected and a smaller deep flap was created to allow access to Schlemm canal. Then, phacoemulsification and lens implantation was performed as described above. Afterwards, the dissection of the deep flap was continued into the clear cornea creating a trabeculo-Descemet window. The deep scleral flap was then removed. A laser-illuminating microcatheter (iTrack™ 250A, iScience Interventional Corporation, Menlo Park, CA, USA) was circumferentially inserted into Schlemm canal. After 360° catheterization, a 10.0 prolene suture was tied to the tip of the microcatheter and pulled back while viscoelastic (Healon GV, Advanced Medical Optics, Inc., Santa Ana, CA) was injected for circumferential dilation of Schlemm canal. The suture was then tightened to permanently stretch the trabecular meshwork. The superficial flap was secured watertight with 10.0 vicryl suture to prevent bleb formation followed by closure of the conjunctiva.

### Postoperative management

#### Phacotrabeculectomy

All patients in the phacotrabeculectomy group received prednisolone acetate eye drops every 2 hours or every hour for one week tapering over 6 to 8 weeks, antibiotic eye drops (gentamycin tid) for one week or as needed, and cycloplegic eye drops (atropine bid) to prevent malignant glaucoma and reduce posterior synechiae in case of increased anterior chamber inflammation for 1 to 2 weeks. In addition to the detailed ophthalmic examination mentioned above, IOP-lowering medication, complications, and postoperative interventions were recorded at each visit. Laser suture lysis was used to control IOP in the early postoperative period when IOP was elevated despite successful outflow after bleb massage. Additionally, intensive postoperative care to control wound healing included increased topical steroid application, early subconjunctival 5-fluorouracil (5-FU) or anti-VEGF injections when bleb scarring was impending, and needling for encapsulated blebs as proposed by Marquardt and coworkers in 2004 [18].

#### Phacocanaloplasty

In contrast to phacotrabeculectomy, the postsurgical regime of patients in the phacocanaloplasty group consisted of prednisolone acetate eye drops every 2 hours, non-steroidal antiinflammatory eye drops tid, and antibiotic eye drops (gentamycin tid) for one week or as needed. No cycloplegic agents were given because pupil dilation may produce an anterior synechiae of the iris to the Descemet window. Neither laser suture lysis was performed nor subconjunctival injections of 5-FU were applied.

The postoperative treatment regime for both groups did not differ between the 3 surgeons. Postoperative visits were scheduled at day 1, 7, week 4, month 3, 6 and 12 or whenever they seemed clinically necessary. All patients had at least a 12-month follow-up, which was considered to be the final outcome. The primary endpoint was the postoperative IOP. Secondary endpoints were visual acuity, frequency and type of complications, need for further surgical interventions, number of topical glaucoma medications, and the success rate based on the following criteria: The surgery was considered complete success if the IOP was 21 mmHg or less and the IOP was reduced by 30% or more to baseline measures (definition 1a) or if the IOP was less than 18 mmHg (definition 2a) without IOP-lowering medication. Qualified success was defined as an IOP of 21 mmHg or less and IOP reduction of more 30% compared to baseline (definition 1b) or an IOP of less than 18 mmHg (definition 2b) with or without medical treatment.

Anti-glaucomatous medications to control IOP were added when laser suture lysis, subconjunctival injections of 5-FU, bleb needling in the phacotrabeculectomy group or Nd:YAG (Neodynium: yttrium aluminium garnet) laser goniopuncture of the Descemet window in patients with phacocanaloplasty failed.

#### Statistics

SPSS© version 19.0 for Windows (SPSS Inc., Chicago, IL, USA) was used for statistical analyses and to create figures. The Shapiro-Wilk test was used to assess normality of distributions. Student´s *t*-test was used for continuous variables with normal distribution. One-way analyses of variance for repeated measurements (ANOVA) were used to examine IOP during follow-up. Differences between groups in single discrete variables without normal distribution were tested for significance using the Mann–Whitney *U*-test. Dependent data without normal distribution was tested with the Wilcoxon signed-rank test. Categorical variables were evaluated with *χ*^2^ test (Fisher´s exact test). Success was evaluated on the basis of Kaplan-Meier cumulative probability (log-rank test). A maximum two-tailed p value of <.05 indicated statistical significance.

## Results

### Baseline data

Thirty-nine eyes of 39 patients were included into the study. Of these, 20 eyes of 20 patients (51.3%) had phacotrabeculectomy and in 19 eyes of 19 patients (48.7%) phacocanaloplasty was performed.

Patients´ characteristics at baseline are summarized in Table 1. No significant difference between the two groups was found for preoperative IOP, age, gender, BCVA, localisation of eye undergoing surgery, type of glaucoma and number of preoperative hypotensive medications. All patients completed the 1-day, 7-day, 4-week, 3-month, 6-month and 12-month follow-up.

**Table 1:** Patients´ characteristics at baseline

	**Phaco trabeculectomy**	**Phaco canaloplasty**	***P value***
n of eyes (%)	20 (51.3)	19 (48.7)	
			
Eye (%)			
Right	9 (45.0)	9 (47.4)	*1.000 *^*a*^
Left	11 (55.0)	10 (52.6)	
Age (years)			
Mean ± SD	73.6 ± 7.5	72.9 ± 5.7	*.743 *^*b*^
Range	59–89	64–81	
Gender (%)			
Male	6 (30.0)	9 (47.4)	*.333 *^*a*^
Female	14 (70.0)	10 (52.6)	
BCVA (logMAR)			
Mean ± SD	0.71 ± 0.87	0.48 ± 0.39	*.835 *^*c*^
Range	0.00–3.00	0.10–1.70	
IOP (mmHg)			
Mean ± SD	30.0 ± 5.3	28.3 ± 4.1	*.272 *^*b*^
Range	22–39	23–37	
n (%) of patients with topical medications	16 (80.0)	17 (89.5)	
n (%) of patients with systemic medications	4 (20.0)	2 (10.5)	
n of topical anti-glaucomatous medications			
Mean ± SD	2.5 ± 1.2	2.8 ± 1.1	*.309 *^*c*^
Range	0–4	0–4	
Type of glaucoma (%)			
Primary open-angle	10 (50.0)	9 (47.4)	*1.000 *^*a*^
Narrow angles	2 (20.0)	1 (11.1)	
Pseudoexfoliative	10 (50.0)	10 (52.6)	
Previous ocular surgery (%)			
None	10 (50.0)	8 (42.1)	*-*
Laser trabeculoplasty	6 (30.0)	6 (31.6)	
Cyclodestructive	2 (10.0)	4 (21.1)	
Laser peripheral iridotomy*	2 (10.0)	1 (5.3)	

Absolute values (percentage), unless stated otherwise. *SD* standard deviation.

*IOP* intraocular pressure, *BCVA* best-corrected visual acuity, *logMAR* log of the minimum angle of resolution, *n* sample size.

^a^*χ*^2^-test (Fisher´s exact test).

^b^ Student´s *t*-test for independent samples with normal distribution (Shapiro-Wilk test was used to assess normality of distributions).

^c^ Mann–Whitney *U*-test without normal distribution.

* Laser peripheral iridotomy was performed in eyes with slightly narrow angles.

### Intraocular pressure results

**Figure 1:**
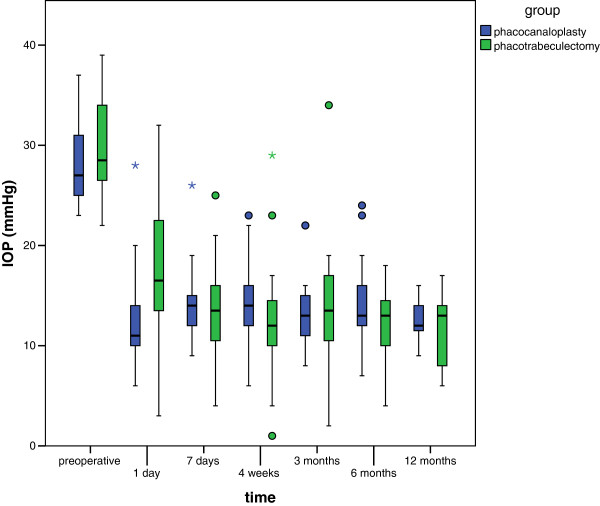
**Intraocular pressure (IOP) outcomes of phacotrabeculectomy and phacocanaloplasty during follow-up.** A highly significant reduction of IOP was found for both groups during follow-up (P < .0001). Except for the first postoperative day, statistical analyses revealed no significant difference for postoperative IOP results between both groups. Box plots illustrate the median (50th percentile) as a black center line and the 25th and 75th percentile as the lower and upper hinges of the box. Circles represent minor outliers and stars major outliers.

**Figure 2:**
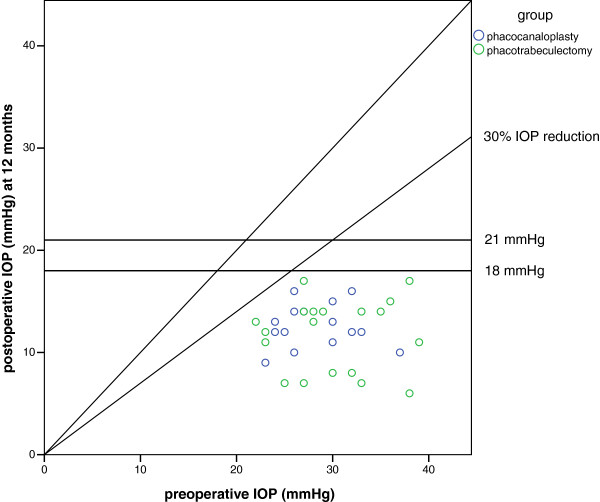
**Scatter plot of preoperative intraocular pressure (IOP) compared to postoperative IOP at the 12-month visit.** Each circle indicates a patient. Points below the oblique line define a lower postoperative IOP than baseline.

At baseline, the mean IOP was 30.0 ± 5.3 mmHg (range 22–39 mmHg) in the phacotrabeculectomy group and 28.3 ± 4.1 mmHg (range 23–37 mmHg) in the phacocanaloplasty group (P = .272). One-way ANOVA for repeated measurements confirmed a highly significant difference between baseline and follow-up within both treatment groups (P < .0001). Mean postoperative IOP was 12.5 ± 6.1 mmHg at 1 month, 13.9 ± 6.4 mmHg at 3 months, 11.9 ± 3.9 mmHg at 6 months and 11.7 ± 3.5 mmHg at 12 months in the phacotrabeculectomy group. Patients in the phacocanaloplasty group had a mean IOP of 14.3 ± 4.2 mmHg at 1 month, 13.5 ± 3.9 mmHg at 3 months, 14.2 ± 4.6 mmHg at 6 months and 12.6 ± 2.1 mmHg at 12 months. Although the extent of IOP reduction was consistently greater in the phacotrabeculectomy group during follow-up, this difference was not statistically significant (Table 2). IOP results of both groups during follow-up are presented in Figure 1. Figure 2 shows the IOP results at 12 month compared to baseline for each patient.

**Table 2:** Intraocular pressure results

	**Phacotrabeculectomy**			**Phacocanaloplasty**			***P value***^***‡***^
	**n**	**IOP (mmHg)**	***P value***^***†***^	**n**	**IOP (mmHg)**	***P value***^***†***^	
Baseline	20	30.0 ± 5.3	***<.0001 ***^***b***^*(n = 19)*	19	28.3 ± 4.1	***<.0001 ***^***b***^*(n = 15)*	*.272*^*a*^
Postoperative							
1 day	20	17.7 ± 7.3		17*	12.8 ± 5.3		***.029***^*a*^
7 days	20	13.6 ± 4.8		17*	14.5 ± 3.9		*.525*^*a*^
4 weeks	20	12.5 ± 6.1		17*	14.3 ± 4.2		*.301*^*a*^
3 months	20	13.9 ± 6.4		17*	13.5 ± 3.9		*.832*^*a*^
6 months	20	11.9 ± 3.9		17*	14.2 ± 4.6		*.111*^*a*^
12 months	19^~^	11.7 ± 3.5		15^+^	12.6 ± 2.1		*.357*^*a*^

Values are means ± SD (standard deviation).

*n* sample size, *IOP* intraocular pressure.

^†^ during follow-up; ^‡^ phacotrabeculectomy versus phacocanaloplasty.

* Two patients developed filtering blebs after canaloplasty and were therefore excluded.

^+^ Two patients needed cyclodestruction to reach target IOP and were therefore excluded.

^~^ One patient underwent cyclophotocoagulation to lower IOP and was therefore excluded.

^a^ Student´s *t*-test for independent samples with normal distribution.

^b^ one-way repeated measures ANOVA.

### Visual acuity results

BCVA was converted to the logarithm of the minimum angle of resolution (logMAR) for statistical analyses. Mean baseline visual acuity was 0.71 ± 0.87 logMAR in the phacotrabeculectomy group and 0.48 ± 0.39 logMAR in the phacocanaloplasty group (P = .835). BCVA was not significantly different between both groups during follow-up. Overall, a mean improvement in visual acuity of 0.19 logMAR in the phacocanaloplasty group and 0.28 logMAR in the phacotrabeculectomy group was seen after 12 months compared to baseline values (Table 3).

**Table 3:** Visual acuity results

	**Phacotrabeculectomy**			**Phacocanaloplasty**			***P value***^***‡***^
	**n**	**BCVA (logMAR)**	***P value***^***†***^	**n**	**BCVA (logMAR)**	***P value***^***†***^	
Baseline	20	0.71 ± 0.87		19	0.48 ± 0.39		*.835 *^*a*^
Postoperative							
1 day	20	1.32 ± 0.95	***<.0001 ***^***b***^	17*	1.17 ± 1.00	***<.0001 ***^***b***^	*.537 *^*a*^
7 days	20	1.14 ± 1.00	***<.0001 ***^***b***^	17*	0.78 ± 0.46	***<.0001 ***^***b***^	*.729 *^*a*^
4 weeks	20	1.02 ± 0.93	***<.0001 ***^***b***^	17*	0.38 ± 0.37	***<.0001 ***^***b***^	***.004 ***^***a***^
3 months	20	0.56 ± 0.66	***<.0001 ***^***b***^	17*	0.28 ± 0.33	***<.0001 ***^***b***^	*.104 *^*a*^
6 months	20	0.40 ± 0.69	***<.0001 ***^***b***^	17*	0.27 ± 0.39	***<.0001 ***^***b***^	*.821*^*a*^
12 months	19^~^	0.43 ± 0.69	***<.0001 ***^***b***^	15^+^	0.29 ± 0.33	***.001 ***^***b***^	*.607 *^*a*^

Values are means ± SD (standard deviation).

*n* sample size, *BCVA* best corrected visual acuity, *logMAR* log of the minimum angle of resolution.

^†^ versus baseline; ^‡^ phacotrabeculectomy versus phacocanaloplasty.

* Two patients developed filtering blebs after canaloplasty and were therefore excluded.

^+^ Two patients needed cyclodestruction to reach target IOP and were therefore excluded.

^~^ One patient underwent cyclophotocoagulation to lower IOP and was therefore excluded.

^a^ Mann–Whitney *U*-test for independent samples without normal distribution.

^b^ Wilcoxon signed-rank test for dependent samples without normal distribution.

### Change in glaucoma medication

Mean number of topical glaucoma medication at the preoperative visit was 2.5 ± 1.2 (range 0 to 4) and 2.8 ± 1.1 (range 0 to 4) in the phacotrabeculectomy and phacocanaloplasty group, respectively. One patient in both groups received only systemic medication at baseline. Preoperatively, 3 patients in the phacotrabeculectomy group and 1 patient in the phacocanaloplasty group needed both systemic and topical IOP-lowering drugs. In the phacocanaloplasty group, the mean number of glaucoma medications was 0.9 ± 1.3 at 6 months and 1.0 ± 1.5 at 12 months. Overall, 7 and 6 patients needed anti-glaucomatous drugs at 6 and 12 months, respectively. In contrast, three patients of the phacotrabeculectomy group were on 1 topical glaucoma drug at 6 months and 4 patients on 1 medication at 12 months. In both treatment groups, the mean number of medications was significantly lower at month 6 and 12 compared to baseline. Mean number of required medication was lower in the phacotrabeculectomy group (Table 4).

**Table 4:** Change in glaucoma medication

	**Phacotrabeculectomy**			**Phacocanaloplasty**			***P value***^***‡***^
	**n**	**Mean number**	***P value***^***†***^	**n**	**Mean number**	***P value***^***†***^	
Baseline	20	2.5 ± 1.2		19	2.8 ± 1.1		*.309 *^*a*^
Postoperative							
1 day	20	0.0	***<.0001 ***^***b***^	17*	0.0	***<.0001 ***^***b***^	*1.000 *^*a*^
7 days	20	0.0	***<.0001 ***^***b***^	17*	0.3 ± 0.8	***<.0001 ***^***b***^	*.557 *^*a*^
4 weeks	20	0.0	***<.0001 ***^***b***^	17*	0.5 ± 1.1	***.001 ***^***b***^	*.232 *^*a*^
3 months	20	0.1 ± 0.3	***<.0001 ***^***b***^	17*	0.7 ± 1.1	***.001 ***^***b***^	*.167 *^*a*^
6 months	20	0.2 ± 0.4	***<.0001 ***^***b***^	17*	0.9 ± 1.3	***.002 ***^***b***^	*.117 *^*a*^
12 months	19^~^	0.2 ± 0.4	***<.0001 ***^***b***^	15^+^	1.0 ± 1.5	***.013 ***^***b***^	*.228 *^*a*^

Values are means ± SD (standard deviation).

*n* sample size.

^†^ versus baseline; ^‡^ phacotrabeculectomy versus phacocanaloplasty.

* Two patients developed filtering blebs after canaloplasty and were therefore excluded.

^+^ Two patients needed cyclodestruction to reach target IOP and were therefore excluded.

^~^ One patient underwent cyclophotocoagulation to lower IOP and was therefore excluded.

^a^ Mann–Whitney *U*-test for independent samples without normal distribution.

^b^ Wilcoxon signed-rank test for dependent samples without normal distribution.

### Success rate

In the phacotrabeculectomy group, success rates were 85.0% (17 patients) based on definition 1a (IOP of ≤21 mmHg and at least 30% reduction of IOP) and definition 2a (IOP of less than 18 mmHg) without IOP-lowering drugs (complete success) at 6 months. At 12 months, success rates were 78.9% (15 patients) for both criteria. 10 eyes (58.8%) and 9 eyes (60.0%) of the phacocanaloplasty group achieved complete success for both criteria after 6 and 12 months, respectively. Although the success rate was lower for patients in the phacocanaloplasty group, statistical analyses did not reveal significant differences of complete success at 6 and 12 months (P > .05; Table 5).

**Table 5:** Success results

	**Phacotrabeculectomy**		**Phacocanaloplasty**		***P value***
	**n**	**Success**	**n**	**Success**	
At 6 months	20		17*		
Complete success					
≤ 21 mmHg + 30% IOP ↓		17 (85.0)		10 (58.8)	*.136 *^*a*^
< 18 mmHg		17 (85.0)		10 (58.8)	*.136 *^*a*^
Qualified success					
≤ 21 mmHg + 30% IOP ↓		20 (100.0)		15 (88.2)	*.204 *^*a*^
< 18 mmHg		19 (95.0)		14 (82.4)	*.315 *^*a*^
At 12 months	19^~^		15^+^		
Complete success					
≤ 21 mmHg + 30% IOP ↓		15 (78.9)		9 (60.0)	*.276 *^*a*^
< 18 mmHg		15 (78.9)		9 (60.0)	*.276 *^*a*^
Qualified success					
≤ 21 mmHg + 30% IOP ↓		19 (100.0)		15 (100.0)	*1.000 *^*a*^
< 18 mmHg		19 (100.0)		15 (100.0)	*1.000 *^*a*^

Absolute values (percentage).

*n* sample size.

* Two patients developed filtering blebs after canaloplasty and were therefore excluded.

^+^ Two patients needed cyclodestruction to reach target IOP and were therefore excluded.

^~^ One patient underwent cyclophotocoagulation to lower IOP and was therefore excluded.

^a^*χ*^2^-test (Fisher´s exact test).

**Figure 3:**
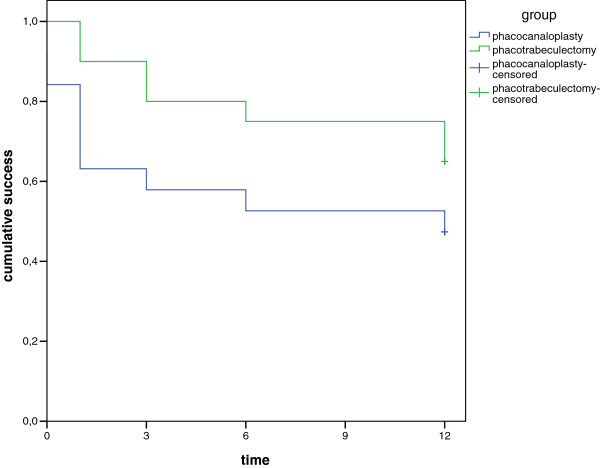
**Kaplan-Meier survival plot for cumulative probability of complete success.** Success was defined as an intraocular pressure (IOP) of less than 18 mmHg without glaucoma medication (complete success). Censored data occurred if a patient completed the 12-month visit and maintained an IOP of less than 18 mmHg without glaucoma medication over 12 months. In the phacocanaloplasty group, the line starts below 1.0 because 2 patients developed a filtering bleb in the early postoperative period and were countered as a failure.

Qualified success was achieved by all 19 patients (100%) of the phacotrabeculectomy group and all 15 patients (100%) of the phacocanaloplasty group having an IOP of less than 18 mmHg, or ≤21 mmHg and at least a 30% reduction of IOP after 12 months (P =1.000). There was no statistically significant difference in qualified success found between both groups (Table 5). Kaplan-Meier survival plots for cumulative probability rates of success defined as an IOP of less than 18 mmHg are shown in Figure 3.

### Incidence of postsurgical complications

The incidence of complications following phacotrabeculectomy and phacocanaloplasty are shown in Table 6.

**Table 6:** Intraoperative and postoperative complications

	**Phacotrabeculectomy**	**Phacocanaloplasty**	P value*
n of eyes (%)	20 (51.3)	19 (48.7)	
*Intraoperative*			
Microperforation TDM	-	0 (0.0)	*-*
Macroperforation TDM	-	1 (5.3)	*-*
*Early postoperative* (≤ 90 days)			
Hypotony (IOP <5 mmHg)			
n	3 (15.0)	0 (0.0)	*.231*
Duration (days)	1–15	-	
Hypotony-related:			
Choroidal detachment	2 (10.0)	0 (0.0)	*.487*
Shallow anterior chamber	1 (5.0)	0 (0.0)	*1.000*
5-FU-related corneal erosion	6 (30.0)	-	*-*
Hyphema ≥ 1 mm layered blood	1 (5.0)	4 (21.1)	*.182*
*Late postoperative* (> 90 days)			
Hypotony (IOP <5 mmHg)	1 (5.0)	0 (0.0)	*1.000*
Choroidal detachment	1 (5.0)	0 (0.0)	*1.000*
Conjunctival leakage	1 (5.0)	0 (0.0)	*1.000*
Bleb formation	-	2 (10.5)	*-*

Absolute values (percentage).

*TDM* trabeculo-Descemet membrane, *n* sample size, *IOP* intraocular pressure, *5-FU* 5-fluorouracil.

^*^χ^2^-test (Fisher´s exact test).

In the phacocanaloplasty group, one patient (5.3%) had an intraoperative perforation of the trabeculo-Descemet membrane. 360° dilation and insertion of a suture into Schlemm canal was successful in all patients with phacocanaloplasty. In one case of the phacotrabeculectomy group, anterior vitrectomy had to be performed and an iris claw lens was placed (5.0%). During the early postoperative period (≤ 90 days), there was less hypotony, defined as IOP of <5 mmHg, in the phacocanaloplasty group than in the phacotrabeculectomy group. Transient hypotony was seen in 3 patients (15.0%) in the phacotrabeculectomy group and in none of the eyes in the phacocanaloplasty group (P = .231). Choroidal detachment occurred in 2 patients (10.0%) with phacotrabeculectomy and in none of the phacocanaloplasty group (P = .487). Hyphema was observed in 4 patients (21.1%) of the phacocanaloplasty group and in 1 patients (5.0%) of the phacotrabeculectomy group (P = .182). During the late postoperative period (> 90 days), 1 patient (5.0%) of the phacotrabeculectomy group had an IOP of less than 5 mmHg and choroidal detachment due to a conjunctival leakage. Two patients (10.5%) with phacocanaloplasty were reported to have a filtering bleb. Postsurgical complications were not found to be significantly different between both groups.

### Bleb management and postsurgical interventions

Table 7 summarizes the postoperative interventions for both treatment groups, bleb management following phacotrabeculectomy and required procedures at the trabeculo-Descemet window in the phacocanaloplasty group.

**Table 7:** Postsurgical interventions and bleb management

	**Phacotrabeculectomy**	**Phacocanaloplasty**	P value*
n of eyes (%)	20 (51.3)	19 (48.7)	
Laser suture lysis			
n of patients	10 (50.0)	-	*-*
Number of sutures	0.8 ± 0.9	-	*-*
5-fluorouracil (5-FU) bleb injections			
n of patients	16 (80.0)	-	*-*
Number of 5-FU injections	6.2 ± 3.7	-	*-*
Range	0–10		
VEGF-inhibitor bleb injections			
n of patients	3 (15.0)	-	*-*
Number of anti-VEGF injections	0.2 ± 0.4	-	*-*
Range	0–1		
Bleb needling	3 (15.0)	-	*-*
Conjunctival suture	1 (5.0)	0 (0.0)	*1.000*
Scleral flap revision	2 (10.0)	-	*-*
Nd:YAG laser goniopuncture	-	2 (10.5)	*-*
Iridoplasty	-	1 (5.3)	*-*
Additional IOP-lowering procedures			
Cyclodestructive	1 (5.0)	2 (10.5)	*.605*

Absolute values (percentage).

*VEGF* vascular endothelial growth factor, *Nd:YAG* Neodynium*:* neodymium yttrium aluminium garnet, *IOP* intraocular pressure, *n* sample size.

^*^χ^2^-test (Fisher´s exact test)*.*

In the phacotrabeculectomy group, laser suture lysis was performed in 10 eyes (50.0%) for patients with increased IOP and flat filtering blebs, which inflated after ocular massage. Subconjunctival injections of 5-FU were repeatedly given in 16 patients of the phacotrabeculectomy group (80.0%; 6.2 ± 3.7 injections of 5-FU, Range 0–10). We additionally applied subconjunctival injections of anti-VEGF in the early postoperative period. In 3 cases (15.0%) of expected increased and rapid bleb scarring, anti-VEGF was given once before starting 5-FU. Bleb needling became necessary in 3 patients (15.0%) of the phacotrabeculectomy group. Nd:YAG goniopuncture of the Descemet window was performed in 2 patients (10.5%) of the phacocanaloplasty group. Further IOP-lowering procedures included cyclodestructive surgeries in 1 patient (5.0%) of the phacotrabeculectomy group and 2 patients (10.5%) of the phacocanaloplasty group (P = .605). No statistically significant difference in postsurgical interventions was found between the two groups.

## Discussion

Combined cataract and glaucoma surgery, both filtering and non-penetrating, has increasingly obtained interest due to the frequent coincidence of both conditions in the elderly population and the frequent occurrence of cataract progression after glaucoma surgery [1-3]. Canaloplasty is a relatively new non-penetrating surgical procedure which aims for restoring the natural aqueous drainage pathway by circumferentially dilating Schlemm canal using a microcatheter to viscodilate and insert a suture into Schlemm canal to keep it permanently open. The major advantage of this non-penetrating surgical method compared to trabeculectomy is that it avoids serious complications associated with filtering blebs. Combining canaloplasty with clear corneal phacoemulsification seems to allow further improvement of the aqueous outflow and provides higher IOP reduction than performed separately due to alterations of the architecture of the angle resulting in a more open configuration [15-17].

This clinical trial analyzed one-year results of phacotrabeculectomy and phacocanaloplasty. The data did not reveal statistically significant differences in IOP control between both groups. Nevertheless, postsurgical complications were more frequent seen after phacotrabeculectomy.

It is well known that cataract surgery alone can provide a reduction of IOP, although the effect is generally small [6,7]. IOP reduction after clear corneal phacoemulsification is considered to be a consequence of increased outflow of aqueous humor due to an increased angle width and tensioning of the trabecular meshwork. Shingleton and coworkers [6] found that IOP reduction was maintained 5 years after cataract surgery. In contrast, IOP is often increased in the early postoperative period in glaucomatous eyes after cataract surgery [6].

Interestingly, several comparative studies on trabeculectomy and phacotrabeculectomy found a higher incidence of postoperative adverse events with the combined procedure. These were mostly induced by an inflammatory response that would limit the efficacy on IOP reduction [2,19,20]. Lochhead et al. [20] reported that phacotrabeculectomy was not as effective as trabeculectomy alone in reducing IOP. A possible mechanism that leads to a smaller IOP reduction following phacotrabeculectomy may be the prolonged anterior chamber inflammation due to an altered blood-aqueous barrier [20].

In contrast to outcomes of combined filtering glaucoma surgeries, studies on non-penetrating glaucoma surgery combined with cataract surgery showed a better IOP-lowering effect of combined techniques [12,13,16]. Bull et al. [16] examined clinical outcomes of canaloplasty compared to phacocanaloplasty and found better results on IOP reduction in combined surgical cases than in cases with canaloplasty alone. In a multicenter-study conducted by Lewis and co-authors [17], 3-year results of canaloplasty and phacocanaloplasty were reported. They concluded that both methods led to a significant and sustained IOP reduction with a low incidence of postoperative complications. Additionally, other clinical trials found a conjunctive effect of non-penetrating glaucoma surgery such as viscocanalostomy [13,14] or deep sclerectomy [11,12], when performed in combination with cataract surgery.

In our study, we found a 12-month IOP of 11.7 ± 3.5 mmHg for eyes undergoing phacotrabeculectomy, which was lower than in patients treated with phacocanaloplasty (12.6 ± 2.1 mmHg), although this difference was not statistically significant (P = .357). Additionally, the phacotrabeculectomy group needed fewer glaucoma medications (0.2 ± 0.4) compared to the phacocanaloplasty group (1.0 ± 1.5) at 1 year (P = .228). These results might be taken into the surgeon´s considerations, if a higher IOP reduction is necessary in patients with advanced glaucoma or if topical glaucoma drugs are not tolerated. Recently, a study by Ayyala et al. [10], comparing surgical outcomes of canaloplasty and trabeculectomy without simultaneous cataract surgery, also found lower IOP with a lower percentage of patients needing postoperative anti-glaucomatous medications for the trabeculectomy group.

In general, studies on combined glaucoma surgeries report a postoperative IOP for phacotrabeculectomy comparable to our results [11,14,20]. For phacocanaloplasty, 1-year outcomes of IOP ranging from 13.7 mmHg to 14.0 mmHg were also comparable to our results [15-17]. Additionally, non-penetrating surgical methods such as deep sclerectomy [11,12] and viscocanalostomy [13,14] in combination with cataract surgery led to similar 1-year results of IOP reduction.

In fact, it is difficult to compare the incidence of complications between a penetrating and a non-penetrating glaucoma procedure. Nevertheless, severe hypotony-related complications are more frequent seen after trabeculectomy. In our study, hypotony (15.0%), choroidal detachment (10.0%) and shallow anterior chamber (5.0%) were seen in the phacotrabeculectomy group whereas in none of the patients in the phacocanaloplasty group. In literature, studies report an incidence of hypotony ranging from 18.5% to 20%, choroidal detachment in the range of 9.3% to 20% and a flattening of the anterior chamber in 0% to 10% following phacotrabeculectomy during 1 to 3 years of follow-up [14,19].

Several studies comparing non-penetrating with traditional incisional glaucoma surgery with or without cataract surgery confirmed lower rates of severe postoperative complications for non-penetrating procedures [8-11,14]. In our study, hyphema was seen in 21.1% of patients with phacocanaloplasty and is a common event after canaloplasty. Hyphema seems to be a positive prognostic value regarding IOP development, since postsurgical interventions to reach target IOP became significantly fewer necessary in patients with a microhyphema [21]. Grieshaber and co-authors concluded that a hyphema may indicate a patent physiologic aqueous drainage system [21]. While dilating Schlemm canal with viscoelastics, a detachment of Descemet membrane may occur. An intracorneal hematoma is a rare complication following Descemet detachment and can be removed by partial-thickness paracentesis [22]. Descemet detachment was not encountered in our study. Overall, complication rate for phacocanaloplasty in our study was low and is comparable to previously published studies [10,15-17]. Bull and coauthors [16] reported a 12.8% incidence of microhyphema, hyphema in 5.5% and Descemet detachment in 3.7%. No hypotony or shallow anterior chamber was seen after canaloplasty and phacocanaloplasty during the early postoperative period [16].

Postoperative bleb manipulation to enhance flow such as focal massage, laser suture lysis, subconjunctival application of antimetabolites and bleb needling is often required after trabeculectomy. Since canaloplasty is a non-filtering, bleb-independent surgical approach, this might be a beneficial advantage compared to trabeculectomy.

Several limitations of this study have to be discussed. This trial was a retrospective and non-randomized; comparison of two separate series commonly having more sources of error due to confounding and selection bias than prospective studies. Additionally, phacotrabeculectomy and phacocanaloplasty were performed by more than one surgeon, though in the same surgical center. Despite the limitation imposed by inclusion of surgical data of different surgeons, both groups had similar preoperative demographic characteristics and the postoperative care was identical for all included patients. Individual predictive factors influencing the long-term outcomes of both combined surgical approaches are needed to decide whether a filtering or non-filtering methods lead to better results.

## Conclusions

In conclusion, phacotrabeculectomy and phacocanaloplasty resulted in a significant reduction of IOP after 12 months. Nevertheless, IOP was higher and more glaucoma medications were required in patients who underwent phacocanaloplasty. Although canaloplasty may not achieve as low pressures as trabeculectomy, combining canaloplasty with cataract surgery may be a valuable alternative to traditional combination of incisional glaucoma surgery and phacoemulsification, since the newer techniques produce moderate IOP reduction comparable to phacotrabeculectomy, have lower rates of postoperative complications and do not need an intensive postoperative care. These results on comparing filtering with non-filtering glaucoma surgery need to be confirmed in prospective randomized studies.

## Abbreviations

IOP: Intraocular pressure; BCVA: Best corrected visual acuity; MMC: mitomycin C; BSS: Balanced salt solution; Phaco: Phacoemulsification; 5-FU: 5-fluorouracil; Nd:YAG: Neodynium: yttrium aluminium garnet; ANOVA: analyses of variance for repeated measurements; logMAR: logarithm of the minimum angle of resolution.

## Competing interests

All authors declare that they have no competing interests.

## Authors’ contributions

JM has collected the data of the trial, designed the study, performed the statistical analysis and drafted the manuscript. FJF collected the data and critically revised the manuscript. SL collected the data and participated in the design of the study. FG performed the surgeries and has given final approval of the version to be published. TK also performed the surgeries, was involved in drafting the manuscript and participated in its design and coordination, and has also given final approval of the version to be published. All authors read and approved the final manuscript.

## Pre-publication history

The pre-publication history for this paper can be accessed here:

http://www.biomedcentral.com/1471-2415/13/1/prepub
